# Polyethylene Glycol 20k. Does It Fluoresce?

**DOI:** 10.1021/acsomega.3c01124

**Published:** 2023-04-06

**Authors:** Bethany F. Laatsch, Michael Brandt, Brianna Finke, Carl J. Fossum, Miles J. Wackett, Harrison R. Lowater, Alex Narkiewicz-Jodko, Christine N. Le, Thao Yang, Elizabeth M. Glogowski, Scott C. Bailey-Hartsel, Sudeep Bhattacharyya, Sanchita Hati

**Affiliations:** † Department of Chemistry and Biochemistry, University of Wisconsin-Eau Claire, Eau Claire, Wisconsin 54701, United States; ‡ Department of Materials Science and Biomedical Engineering, University of Wisconsin-Eau Claire, Eau Claire, Wisconsin, 54701, United States

## Abstract

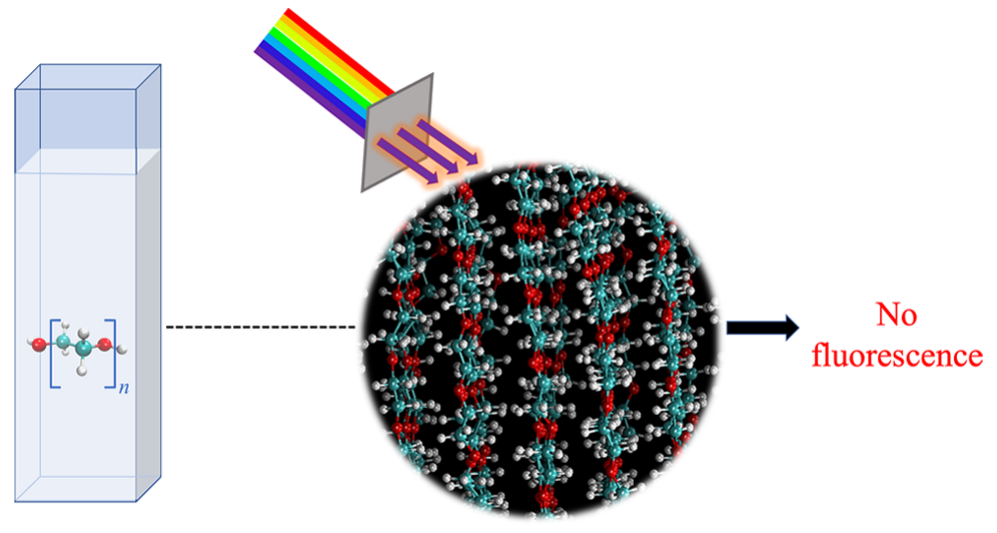

Polyethylene glycol (PEG) is a polyether compound commonly used in biological research and medicine because it is biologically inert. This simple polymer exists in variable chain lengths (and molecular weights). As they are devoid of any contiguous π-system, PEGs are expected to lack fluorescence properties. However, recent studies suggested the occurrence of fluorescence properties in non-traditional fluorophores like PEGs. Herein, a thorough investigation has been conducted to explore if PEG 20k fluoresces. Results of this combined experimental and computational study suggested that although PEG 20k could exhibit “through-space” delocalization of lone pairs of electrons in aggregates/clusters, formed via intermolecular and intramolecular interactions, the actual contributor of fluorescence between 300 and 400 nm is the stabilizer molecule, i.e., 3-*tert*-butyl-4-hydroxyanisole present in the commercially available PEG 20k. Therefore, the reported fluorescence properties of PEG should be taken with a grain of salt, warranting further investigation.

## Introduction

Polyethylene glycol (PEG) is a flexible, non-toxic polymer.^1,2^ Due to its repeating ether groups, low-molecular-weight PEGs are hydrophilic and high-molecular-weight PEGs are amphiphilic in nature.^3,4^ PEG is considered biologically inert and has numerous applications in medicine and industry. One common application of PEG is as an osmotic laxative.^5^ Also, PEG is often attached to drug molecules in a process called PEGylation to enhance their stability and solubility, decrease the immune response, and increase circulation time throughout the body.^6^ Recently, PEGylated lipids have been included as an ingredient in the vaccines for COVID-19, and PEG allergies in patients are suspected to be the likely cause of the rare anaphylactic response to the vaccine.^3^ Additionally, PEG is used to coat surfaces to serve as a lubricant or a preservative.^7,8^ Moreover, PEG molecules of variable sizes are commonly used for studying the effects of molecular crowding and confinement on the conformation and function of proteins and nucleic acids.^9,10^ While probing the conformational change of a protein in crowded “intracellular-like” environments using intrinsic fluorescence spectroscopy, it was observed that the solution crowded with PEG of 20 kDa molecular weight (PEG 20k) fluoresced even in the absence of the protein under investigation; the fluorescence intensity of an aqueous solution containing phosphate buffer, PEG 20k, and sodium chloride was abnormally high. Unlike traditional fluorophores, which possess conjugated π-systems, PEG does not have such structural characteristics but interestingly fluoresced regardless.

Based on recent studies, it was revealed that PEG can be classified as a nontraditional intrinsic luminophore, and the resulting luminescent properties are due to the phenomenon referred to as aggregation-induced emission (AIE).^4,11−14^ When in an aggregated state, heteroatoms are brought into proximity so that their lone pairs of electrons are easily delocalized. Additionally, the aggregation creates a more rigid structure, thereby reducing non-radiative relaxation.^15^ In a recent study, the PEG of molecular weights from 2k to 12k were found to be emissive.^14^ Moreover, it was reported that PEG 8k has the maximum emission among PEG 2k to 12k at 340 nm when excited at 280 nm. The fluorescence property of PEG 20k, however, remained unexplored. Various experimental studies revealed aggregate formation that might induce intrinsic fluorescence properties to PEG molecules via “through-space” delocalization.^14^ However, the exact mechanism of the fluorescence has remained elusive. In the present study, we report a comprehensive investigation of the molecular basis of the fluorescent properties of PEG 20k molecules using combined experimental and computational methods. Fluorescence measurements of PEG 20k were conducted in aqueous solutions as well as in the presence of metal ions. Also, atomic force microscopy (AFM), dynamic light scattering (DLS), fluorescence anisotropy, and proton nuclear magnetic resonance (^1^H NMR) were used to analyze PEG 20k aggregates, assess their sizes, and investigate the presence of delocalization. Atomistic molecular dynamics (MD) simulations were performed to explore the structural properties of PEG 20k. Quantum chemical calculations were performed at the level of density functional theory with improved functionals^16−18^ to probe if the “through-space” delocalization of the PEG 20k aggregates gives rise to the fluorescence properties.

## Methods

### Experimental Section

#### Fluorescence Spectroscopy

Steady-state fluorescence experiments were performed using an Agilent Cary Eclipse fluorescence spectrophotometer. Samples of various concentrations of PEG 20k and other variable-sized PEG molecules were prepared by dissolving the required quantity of PEG in water or water along with 100 mM NaCl and 30 mM phosphate buffer of pH 7.4 to mimic biological conditions. Solutions of 100 mg/mL PEG 20k in the presence of six different metal ions were prepared using 10 mM NaCl, MgCl_2_, CoCl_2_, FeCl_3_, CrCl_3_, and K_2_CrO_4_. Blank samples containing only water and the corresponding metal salt were prepared for each of the six ionic compounds. PEG 600, PEG 2k, and PEG 8k were purchased from Thermo Fisher Scientific and were used as received without further purification. PEG 20k was obtained from Thermo Fisher Scientific and MilliporeSigma. Fluorescence of PEG 20k was recorded before and after purification with diethyl ether. Samples were excited within a range of 210–300 nm, and the emission spectra were recorded from 300 to 400 nm with 5 mm spectral slits. All experiments were performed using a quartz cuvette with a 1 cm optical path length and were done in triplicate.

#### Gas Chromatography–Mass Spectrometry

13 mL of 400 mg/mL PEG 20k sample (purified before and after washing with diethyl ether 6 and 12 times) was pipetted from the original sample into a 40 mL brown septum vial with a lid, and a Monotrap monolithic disc coated with octadecyl silane activated carbon (Monotrap DCC18, GL Sciences) was floated on top. Samples were then incubated in a water bath at 37 °C for 1 h. Following removal from the water bath, the Monotrap disk was removed from the vial, patted dry with a Kimwipe, and gently placed into a 3.0 mL microreaction vial with a lid. 2.0 mL of mass spectrometry (MS)-grade dichloromethane (DCM) was added, and the lid was tightly shut. The samples were put into the sonication bath for 10 min. All the remaining DCM was then extracted from the microreaction vial and placed into 1.5 mL Agilent screw cap vials and labeled. The compounds were separated and detected using an Agilent Technologies gas chromatography system (model: 7820 A GC System) with a quadrupole mass spectrometer. The instrument used a J&W DB624 GC column, 60 m, 0.32 mm, 1.80 μm, 7 inches. The oven temperature was set to 40 °C for 3 min and then increased to 320 °C at a rate of 9 °C/min. The front inlet temperature was 250 °C. The injection was in the split mode with a 1:1 ratio and 1 μL of injection volume at 7.14 psi. The detector temperature was 230 °C. The detection and data acquisition were performed in the scan mode at 35 scans/s, from 35 to 450 Da. Peaks were identified by comparison with the National Institute for Standards and Technology (NIST) database. All samples were done in duplicate, and a deionized H_2_O control was run alongside the PEG 20k duplicates.

#### Atomic Force Microscopy

Samples were prepared by applying 100 μg/mL, 20 mg/mL, 200 mg/mL, and 300 mg/mL PEG 20k solutions to freshly cleaved pieces of highly oriented pyrolytic graphite (HOPG) as to cover the whole surface of the HOPG. Samples on HOPG were incubated for 30 min. After 30 min, excess PEG 20k solution was removed from the HOPG substrate with a dry Kimwipe by capillary action. The sample was then dried in vacuo for 30 min and scanned using the MFP-3D Origin atomic force microscope from Asylum Research. Scans were conducted in a 5 μm area using the uniqprobe qp-BioAC probe and a force constant of 0.15–0.55 N/m. The scan rate was maintained at 0.4 Hz, and the scans were done with 512 scan points and lines. All samples were run in duplicate.

#### Dynamic Light Scattering

To examine if there is a formation of vesicles of PEG 20k in water and assess the distribution of the hydrodynamic radius of those vesicles, the DLS experiment was conducted using a Wyatt Mobius in the DLS-only mode with disposable cuvettes at 20 °C. Standard correlation cutoffs were used with auto-attenuation on and normal laser mode with a laser wavelength of 532.0 nm and a detector angle of 163.5°. An acquisition time of 2 s was used, and 10 runs were averaged per sample set. Each sample was duplicated 5 times to determine the statistical consistency of the data. The built-in Wyatt-developed regularization fit was used to determine the average hydrodynamic radius assuming multiple peaks in solution with data plotted in radius and percent intensity with a random coil model. Samples of 100, 200, and 300 mg/mL PEG 20k were each prepared with 100 mM NaCl and 10 mM phosphate buffer, pH 7.4, and filtered using VWR brand PES membrane 0.2 μm syringe filters.

#### Fluorescence Anisotropy

Anisotropy can reveal information about the size, shape, and rigidity of a sample in solution. Samples of 100, 200, and 300 mg/mL PEG 20k were each prepared in 100 mM NaCl and 10 mM phosphate buffer, pH 7.4. The anisotropy measurements were conducted with the automated polarizer accessory of the Agilent Cary Eclipse fluorescence spectrophotometer. Samples were excited at 288 nm because it was found to give the highest emission intensity recorded from 320 to 340 nm at temperatures of 25 and 50 °C.

#### 1D-^1^H NMR

The NMR experiment was conducted to analyze the chemical shift of the ethylene group of PEG 20k in D_2_O at different concentrations. Solid PEG 20k was dissolved in D_2_O to prepare 20 and 200 mg/mL solution. 1D-^1^H NMR spectra were recorded using the Bruker Avance II 400 MHz NMR spectrometer.

#### UV–Vis Spectroscopy

The samples (30 and 300 mg/mL of PEG 20k) were prepared by dissolving the required amount of solid PEG 20k in deionized water. The spectra were recorded from 190 to 900 nm using the Varian Cary 50 UV–Vis spectrophotometer.

### Computational

#### Software and Hardware

Visualization and molecular editing were carried out using the visual MD (VMD) program suite.^19^ All MD simulations were performed using GPU-enabled nanoscale MD (NAMD).^20,21^ Calculations were performed on the hybrid GPU–CPU BOSE supercomputer with 61 nodes and 3904 cores of the Blugold Center for High-Performance Computing, University of Wisconsin-Eau Claire. Each node of the cluster contains two CPUs, equipped with 2.3 GHz/32-core AMD EPYC 7452, and is connected through Hewlett Packard Slingshot internode connection. Additionally, GPU nodes have NVIDIA Tesla V100S 32GB GPU cards that enable NAMD to have hardware acceleration using GPU or CPU vectorization.

The CHARMM36 force field^22,23^ was employed for molecular mechanical calculations. Non-bonding interactions were modeled using a switching function with a “switchdist” of 10 Å, a cutoff of 14 Å, and a “pairlistdist” of 16 Å. Furthermore, electrostatic interactions were treated with the particle mesh Ewald method.^24^ The leapfrog Verlet algorithm^25^ with a time step of 2 fs was used to compute atomic velocities and displacements. A modified Nosé-Hoover method^26,27^ was employed for conformational sampling, where fluctuations in the barostat were controlled using Langevin dynamics.^28,29^ For all solvated calculations, a periodic boundary condition, which controls the pressure by dynamically adjusting the unit cell volume and rescaling atomic coordinates, was used.

Electronic structure calculations were carried out using density functional theory.^30,31^ Gas- and solvation-phase calculations were performed on Gaussian16 with the M06-2X density functional^16^ using the 6-31+G(d,p) basis set.^32^ Implicit solvation calculations were performed using the continuum solvation model, SMD.^33^ Time-dependent density functional theory (TD-DFT)^34^ was used to compute the excited state and model the electronic absorption of the PEG clusters as well as the monomeric EG. In these calculations, ground-state geometrically optimized structures were used to compute the excited-state potential energies using the Coulomb-attenuating method Becke 3-parameter, Lee–Yang–Parr (CAM-B3LYP) functional^34−37^ along with the 6-311++g(d,p) basis set.^32^

#### Building a PEG 20k Monomer

A PEG 20k molecule was constructed with the CHARMM program^22^ suite using homemade scripts. At first, ethylene glycol (EG) and its dimer were built. A major axis along the dimeric EG unit was defined, and that unit was copied and translated along the axis several times. These units were patched together using a homemade script to build a large chain. The final PEG 20k molecule had a molecular weight of 19,963 Da. The linear construct of the PEG chain was subjected to 100 ns MD simulation. A compact structure of PEG 20k was formed, which was solvated, and the resultant system was minimized for 50 ps. This minimized system was subjected to MD simulation for an additional 100 ns.

#### Building a PEG 20k Heptamer

The linear PEG 20k structure was duplicated 6 times to form a heptamer. The heptamer was first minimized, and then, a 200 ns MD simulation was performed in the gas phase. Upon gas-phase equilibration, the heptameric system was solvated at neutral pH. This resultant system was simulated for an additional 200 ns.

#### Determining the Ground- and Excited-State Structures of PEG 20k Clusters

To examine the structure, electron delocalization, and bonding of the PEG 20k interior, a quantum chemical study was conducted at the level of density functional theory.^30,31^ Briefly, spherical regions of 5, 7.5, and 10 Å radii were extracted from the classical mechanically equilibrated structures and stored in the MD simulation trajectory of the PEG 20K monomer. Hydrogen atoms were added to complete the valences of the carbon and oxygen atoms. The smaller clusters were geometry-optimized using the smaller basis set 6-31+g(d,p). Excited states were computed from these optimized ground-state geometries using the expanded basis set of 6-311++g(d,p). The excited states for the cluster of 10 Å radius could not be determined because of the lack of convergence. A separate calculation of atomic charges was carried out on the ground states using natural bond orbital population analysis, which computes the localized electron density from the optimized electronic wavefunctions.^38^

## Results and Discussion

### Fluorescence Measurements

#### Excitation Wavelength Variation Study of PEG 20k

**Figure 1:**
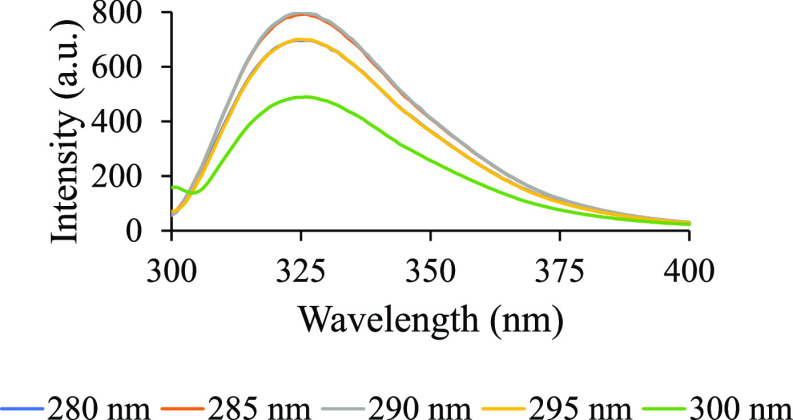
100 mg/mL PEG 20k was excited at wavelengths of 280–300 nm in increments of 5 nm to determine the maximum emissions. Each fluorescence spectrum represents an average of three trials.

The wavelength of maximum emission was examined by measuring the fluorescence of PEG 20k for a range of excitation wavelengths (λ_ex_) (Figure 1). The aqueous solution of unpurified PEG 20k was found to fluoresce at all wavelengths within the range, but the emission intensity was the highest at λ_ex_ ∼ 290 nm for the intervals chosen. In the present study, we decided to use a λ_ex_ of 295 nm due to its significance in protein studies. It should be noted that a comparison of PEG 20k (100 mg/mL) dissolved in buffer (100 mM NaCl and 10 mM phosphate buffer, pH 7.4) versus dissolved in water alone did not display a significant difference in emission intensity. Also, PEG 20k from Thermo Fisher Scientific and MilliporeSigma exhibited similar fluorescence behavior (Figure S1).

#### Fluorescence Behavior of Different Molecular Weights of PEG

**Figure 2:**
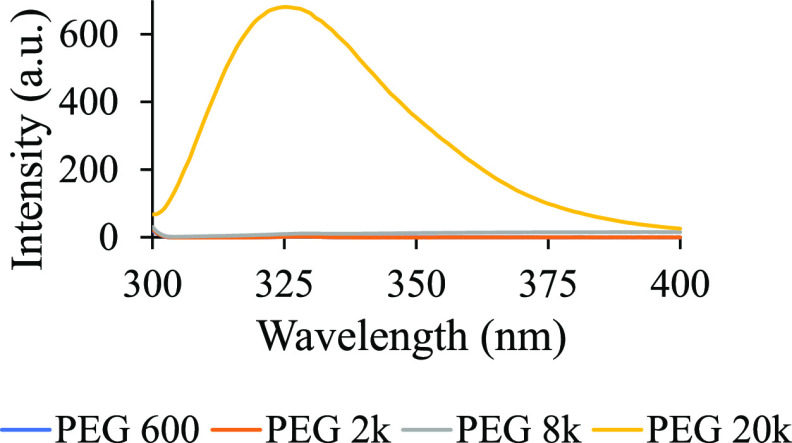
Solutions containing 100 mg/mL PEG, 100 mM NaCl, and 30 mM phosphate buffer of pH 7.4 were excited at 295 nm for molecular weights of PEG 600, PEG 2k, PEG 8k, and PEG 20k. Each fluorescence spectrum represents an average of three trials. No significant fluorescence was observed for any other PEG molecules except for PEG 20k.

The fluorescent properties of PEG of various molecular weights were compared by exciting the PEG samples at 295 nm. Only PEG 20k displayed a significant fluorescence under the experimental condition, i.e., 100 mg/mL PEG in the presence of 100 mM NaCl and 10 mM phosphate buffer, pH 7.4, and λ_ex_ = 295 nm (Figure 2). This was a deviation from the existing literature where PEG 2k to PEG 12k were reported to fluoresce at varying degrees.^4,14^ The earlier work by Sun et al.^14^ revealed an absence of a linear trend between the molecular weight and the extent of emission; PEG 8k exhibited the highest emission intensity when excited at 280 nm, whereas PEG 2k and PEG 12k exhibited very low emission intensity. As a significantly higher fluorescence intensity was observed for PEG 20k and almost no emission was observed from the lower molecular weight PEGs (Figures 2 and S2), it led us to investigate if any chemicals were present as impurities in the commercially available PEG 20k.

### Detection of Impurity Using GC–MS

#### GC–MS experiment to investigate the presence of any impurities in PEG 20k

**Figure 3:**
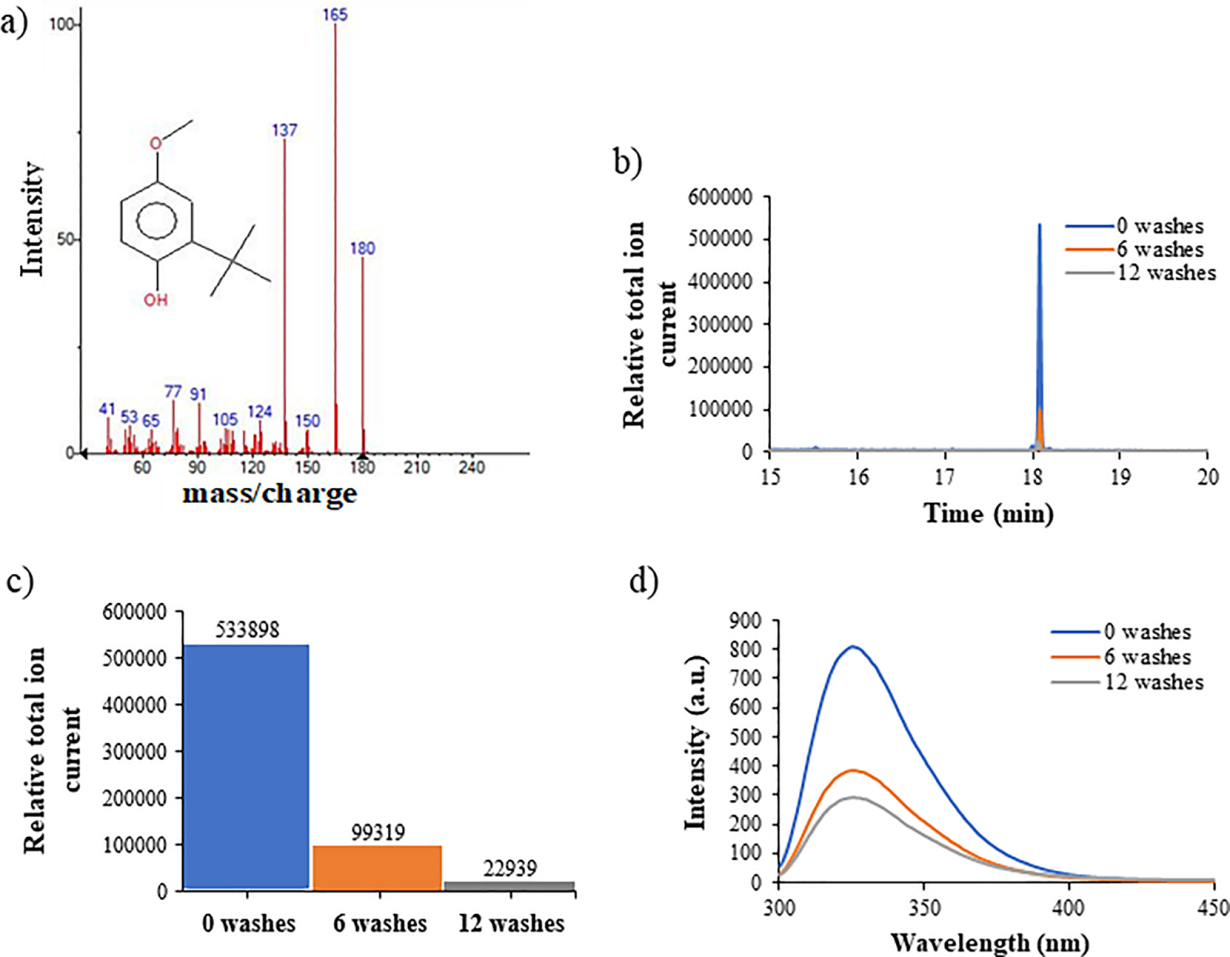
(a) GC–MS spectrum of 3-*tert*-butyl-4-hydroxyanisole (3-BHA), which is a 96% match to the spectrum of 3-BHA from the NIST Chemistry Webbook. (b) Chromatogram of the 3-BHA peak under three different washing conditions of 400 mg/mL PEG 20k. A full 30 min GC–MS analysis was run, and the 3-BHA peak is seen at approximately 18 min. Chromatogram only shows 15–20 min for clarity. (c) Peak intensity for the three trials of PEG 20k with different levels of purity. Data were taken at the apex of each 3-BHA peak, all within 0.02 s of each other and showing a match to 3-BHA in the NIST database. (d) Fluorescence spectra of 100 mg/mL PEG 20k before and after washing with diethyl ether.

To examine the presence of impurities in PEG 20k samples, gas chromatography (GC)–MS experiments were carried out. For the unpurified PEG 20k, a single peak was observed at approximately 18 min in the GC–MS chromatogram (Figure 3). After a comparison of the spectra to the NIST database, a 96% match to the spectra for 3-*tert*-butyl-4-hydroxyanisole (3-BHA) was observed (Figure 3a). A value of 533898 was seen for the total ion current for this trial (Figure 3b,c). The second trial also showed a peak at the same 18 min mark and similar high spectral match values for 3-BHA in the NIST database. The GC–MS chromatograms of PEG 20k after washing with diethyl ether 6 and 12 times were also recorded. Washing with diethyl ether was done by adding an excess of diethyl ether to the powdered PEG 20k and incubating it for 30 min in a closed container. The dissolved 3-BHA in diethyl ether was then extracted. This process was repeated several times. The GC–MS chromatograms of purified PEG 20k demonstrated a significant decrease in peak height compared to the unpurified sample, 81.4% after 6 washes and 95.7% after 12 washes (Figure 3b,c and Table 1). The analysis of the area under the peak for GC–MS spectra revealed a ∼93% decrease in the concentration of 3-BHA after 12 washes with diethyl ether (Table 1). These results suggested that the thorough washing of solid PEG 20k with diethyl ether failed to remove completely the impurities in the form of 3-BHA, and possibly, remaining 3-BHA molecules were encapsulated by the PEG 20k polymer. The fluorescence spectra of PEG 20k (100 mg/mL) revealed a ∼50% decrease in fluorescence intensity after 6 washes and another 10% decrease (a total of 62% decrease) after 12 washes with diethyl ether compared to the unwashed sample (Figure 3d and Table 1). This demonstrates that the percent decrease in the area under fluorescence peaks between 6 and 12 washes is roughly proportional to the decrease in the area under GC–MS peak height between 6 and 12 washes. This correlation and the reduction in fluorescence intensity after treating the solid PEG 20k with diethyl ether suggested that the main contributor to the fluorescence between 300 and 400 nm is 3-BHA. Further investigation revealed that stabilizers like 3-BHA are used for higher molecular weight PEG (Sigma-Aldrich, CAS-No: 25322-68-3).

Although the GC–MS method employed here is qualitative in nature, ∼30 to 40% residual fluorescence intensity does not completely eliminate the possibility of PEG 20k having fluorescent properties. Particularly, previous studies reported the fluorescent properties of various PEG molecules.^4,14^ Therefore, further studies were conducted to examine the possibility of PEG 20k to fluoresce, especially focusing on the aggregate formation and the “through-space” delocalization of lone pairs of electrons on oxygen atoms, resulting in AIE. An attempt was made to examine if PEG 20K forms clusters in an aqueous solution using AFM and DLS.

### Aggregate Formation of PEG 20k

#### AFM Imaging-Identified Small Clusters

**Figure 4:**
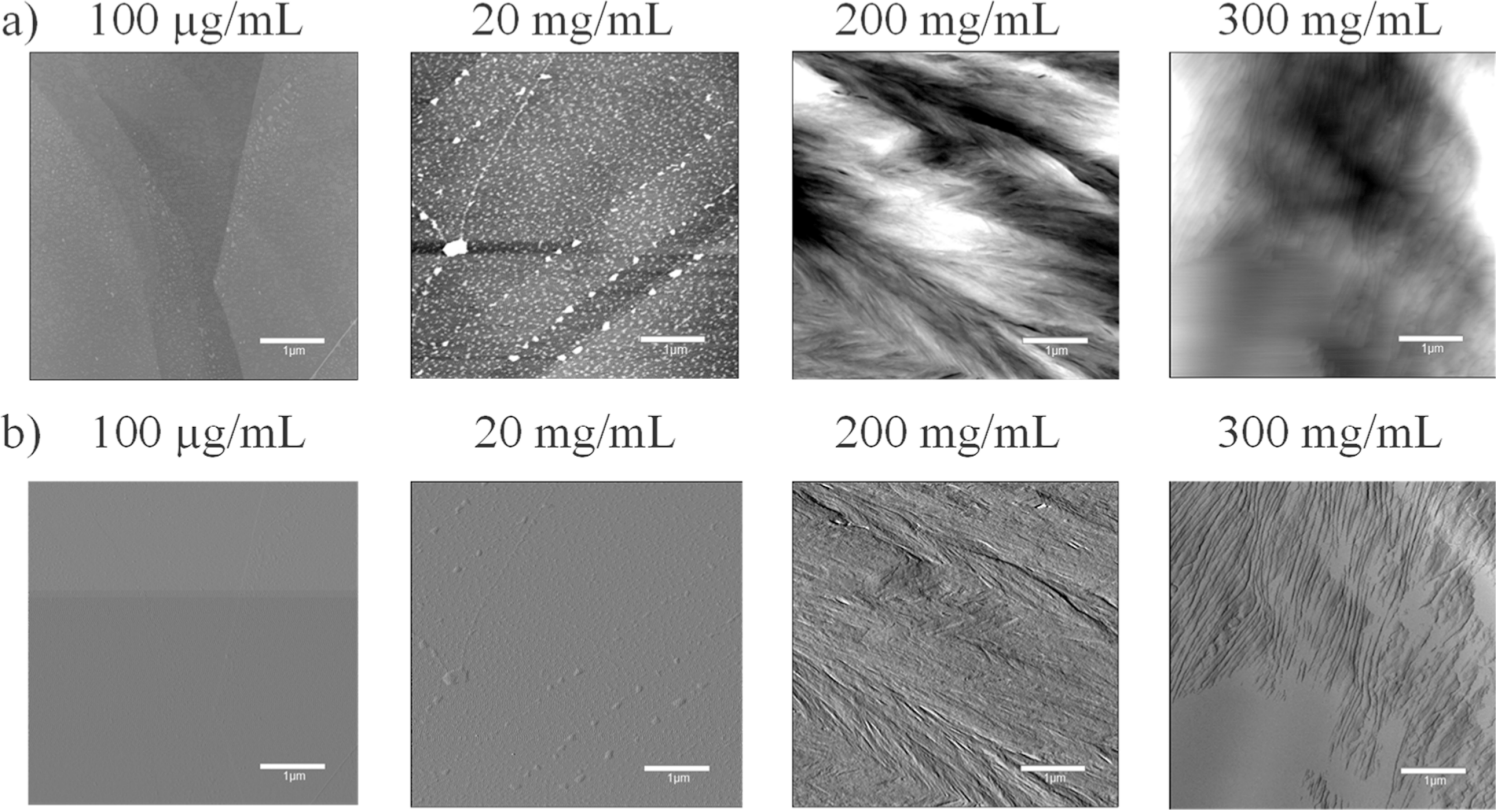
AFM images of the PEG 20 k on the HOPG substrate. (a) Height-retraced and (b) amplitude-retraced images of PEG 20k on the HOPG substrate. Each AFM image size is 5 μm × 5 μm. The key in each image indicates a length of 1 μm. The image of the free HOPG substrate is shown in Figure S3.

Aqueous solutions of PEG 20k of variable concentrations were applied to freshly cleaved pieces of HOPG (free HOPG substrate is shown in Figure S3). The topography of PEG 20k, deposited on the HOPG surface, was investigated by tapping-mode AFM. AFM images exhibited irregular-shaped aggregates (Figure 4). PEG 20k tends to form clusters at all concentrations, but their specific aggregation behavior changes depending on the concentration. At higher concentrations, PEG 20K appeared to aggregate in sheets as observed in the 200 and 300 mg/mL samples. At lower concentrations, PEG clusters formed, but the clusters were separated and smaller in size, as seen in the 20 mg/mL sample. This aggregation behavior was observed even at a lower concentration (100 μg/mL); however, the clusters were tiny.

#### DLS Confirmed the Presence of Small Clusters of PEG 20k

**Figure 5:**
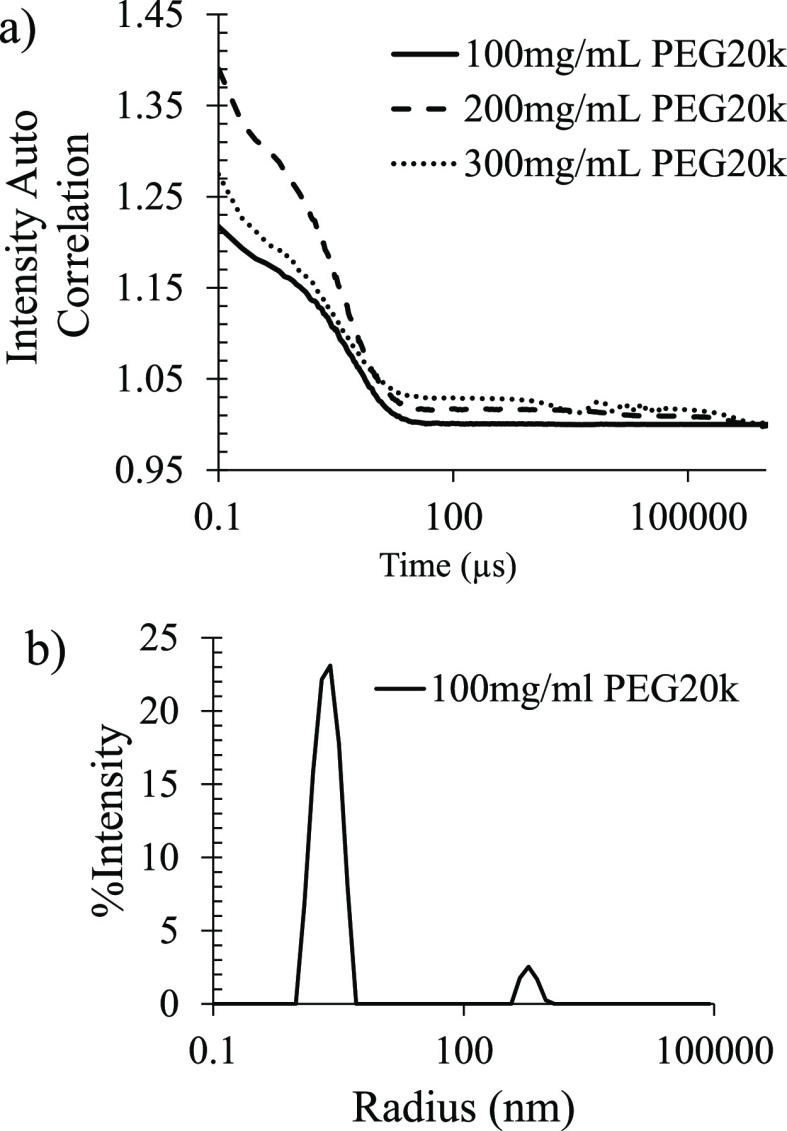
(a) DLS autocorrelation functions of PEG 20k of 100, 200, and 300 mg/mL dissolved in 100 mM NaCl and 30 mM phosphate buffer of pH 7.4. (b) Regularization fit for 100 mg/mL PEG 20k dissolved in 100 mM NaCl and 30 mM phosphate buffer of pH 7.4.

To investigate the average hydrodynamic radius and size distribution of PEG species in solution, DLS analyses were conducted for PEG 20k solutions as a function of polymer concentration. Samples were filtered using PES membrane 0.2 μm syringe filters to ensure no dust or other large contaminants. The autocorrelation data (Figure 5a) showed a plateau that began at approximately 50 μs. This plateau was consistent for all PEG 20k polymer samples and increased in intensity with increasing polymer concentration, which indicated that this was a feature of the sample and not an anomalous result due to low concentration. The regularization fit of the data using a random coil model (Figure 5b) indicated that two species were present. The first peak is centered at 2.5 nm hydrodynamic radius, which corresponds to individual PEG chains. The second peak is centered at 600 nm hydrodynamic radius, which corresponds to clusters of polymer chains. This size range between 10 and 20%, similar to the dispersity for the individual polymer chain’s peak, indicates clearly defined clusters. The clusters are larger than previously reported polymer clusters,^14^ which is consistent with the higher molecular weight PEG being used in this study.

**Table 1:** Comparative Analysis of the Decrease in Fluorescence and GC–MS Peak Areas^a^

	Number of Washes			% Decrease After a Certain Number of Washes	
	0	6	12	6	12
GC–MS peak height	533898	99319	22939	81.4	95.7
the area under the GC–MS peak integrated from 17.95 to 18.25 min	17473	3630	1250	79.2	92.8
the area under the fluorescence peak integrated from 300 to 450 nm	37121	18203	14196	51.0	61.8

a The comparison was made by computing the peak areas for both measurement types. The GC–MS and fluorescence results were obtained after washing PEG 20k with diethyl ether.

#### Fluorescence Anisotropy Measurement to Assess the Cluster Size

**Figure 6:**
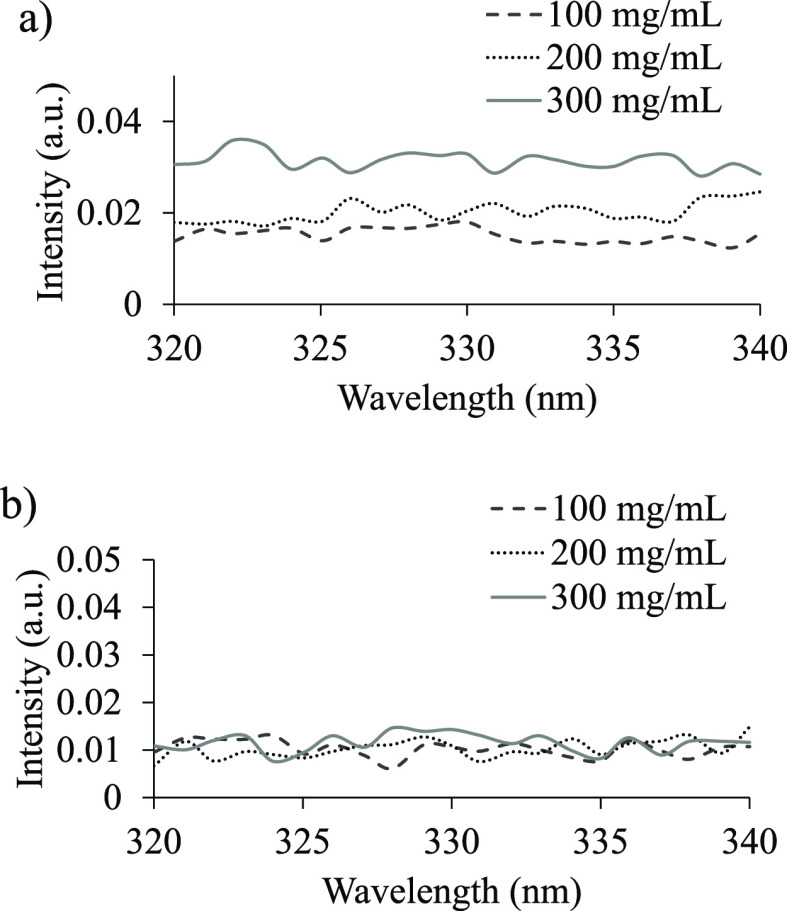
Fluorescence anisotropy of PEG 20k of 100, 200, and 300 mg/mL dissolved in 100 mM NaCl and 30 mM phosphate buffer of pH 7.4. Samples were excited at 288 nm, and emissions were read from 320 to 340 nm. (a) Anisotropy at 25 °C and (b) anisotropy at 50 °C.

Both AFM and DLS experiments suggested the formation of aggregates in an aqueous solution. To examine the size, shape, and rigidity of the PEG 20k in an aqueous solution, fluorescence anisotropy was measured at various concentrations and at different temperatures. A fluorophore with low mobility and slow rotation relative to its fluorescent excitation lifetime will have a larger value of vertically polarized emissions than horizontal and so the anisotropy will be large. A fluorophore with high mobility will tumble rapidly through the solution relative to its fluorescent excitation lifetime and will have similar values of vertically and horizontally polarized emissions, making the anisotropy value small. The samples of 100, 200, and 300 mg/mL PEG 20k were excited at 288 nm, the wavelength at which emission was maximum for PEG 20k, and emissions were read at 320–340 nm at 25 and 50 °C (Figure 6). The anisotropy is higher at 25 °C than at 50 °C for all concentrations of PEG 20k, which is expected as the PEG 20k solution will be more mobile as the temperature and energy of the system increase.

Even at 25 °C, all concentrations of PEG 20k have relatively low anisotropy values. However, the data show a trend that as the concentration increases, so does the anisotropy value (Figure 6a). This suggests that there is less mobility of the PEG 20k aggregates at higher concentrations.

Perhaps this is because, at lower concentrations, there are smaller aggregates of PEG 20k that can easily tumble through the solution, whereas, at higher concentrations, there are larger aggregates that are not as mobile. The expected trends emerged, such as higher anisotropy values at higher concentrations, indicating the presence of some larger aggregates that continue to fluoresce but with a larger hydrodynamic radius. On the other hand, higher temperatures induced lower anisotropy values, indicating that aggregates have dissociated, and fluorescent species are similar in fluorescence properties and likely of similar sizes. Similar anisotropies for a low and high concentration of PEG 20k at a higher temperature (Figure 6b) also implicated the presence of a core structure with a generally low anisotropy value. However, it should be noted that all anisotropy values were relatively low, and there was more depolarization than expected for this relatively large polymer at all temperatures. These low anisotropy values indicated that the observed fluorescence from an aqueous solution of PEG 20k could simply be originating from the small fluorescent impurity, i.e., 3-BHA instead.

#### Melting and Cooling Experiments to Ascertain the Cluster Formation

**Figure 7:**
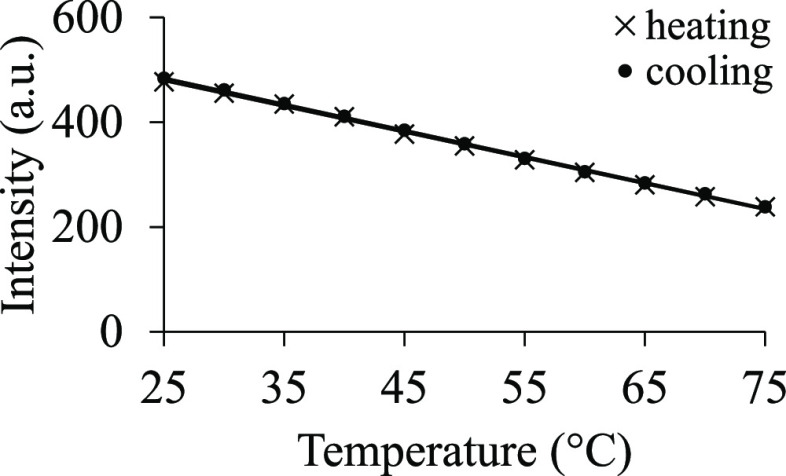
Solution containing 100 mg/mL PEG 20k, 100 mM NaCl, and 30 mM phosphate buffer of pH 7.4 was excited at 295 nm. The emission was recorded at 320 nm. The temperature was increased at 5° increments from 25 to 75 °C (marked with X). Similarly, cooling was done at 5° increments from 75 to 25 °C (marked with filled circles).

In order to understand more about the characteristics of the PEG 20k clusters, a thermal variation experiment was performed in which the fluorescence intensity of the 100 mg/mL PEG 20k solution was measured with increasing temperature. It was found that the fluorescence intensity decreased linearly with increasing temperature (Figure 7). To examine whether the fluorophore could regain its emission intensity and therefore presumably attain its native clustered/aggregated form after cooling, the temperature was subsequently decreased in identical increments. It was found that the melting and cooling processes were reversible. However, no cooperativity in the melting or folding process was observed. This observation indicates two possibilities—(i) the fluorescence intensity decreases at higher temperature due to the disruption in the self-assembled clusters of PEG 20k, which prevented the “through-space” delocalization of lone pairs of electrons on oxygen atoms, and (ii) this decrease in fluorescence intensity is resulting from the dynamic quenching of the 3-BHA, trapped within the PEG 20k matrix, which increases at a higher temperature.

### Fluorescence Quenching due to Metal Ions

#### Impact of Metal ions on PEG 20k Emission Properties

**Figure 8:**
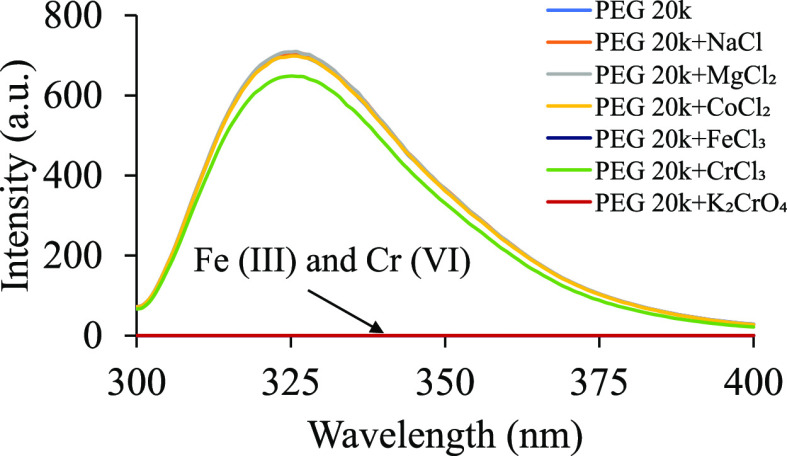
Solutions of 100 mg/mL PEG 20k in the presence of metal ions of 10 mM concentration were excited at 295 nm. Each fluorescence spectrum represents an average of three trials. Na^+^, Mg^2+^, and Co^2+^ had no effect on the fluorescence of PEG 20k. Cr^3+^ displayed minimal quenching, whereas Fe^3+^ and Cr^6+^ quenched the fluorescence of PEG 20k completely.

Metal ions are known to act as fluorescence quenchers by employing different mechanisms such as ground-state complex formation, heavy atom quenching, the paramagnetic effect, resonance energy transfer, etc.^39^ For a specific fluorophore, the quenching mechanism would be unique, and therefore, if the fluorescence properties of PEG 20k are a result of trapped 3-BHA, we would be able to see a pattern of fluorescence quenching, comparable to the free 3-BHA. To test this hypothesis, the fluorescence of PEG 20k was measured in the presence of various metal ions at a ratio of two metal ions for one PEG molecule and was excited at 295 nm. Co^2+^ and Cr^3+^ were observed to quench the fluorescence of PEG 20k to some extent, but Fe^3+^ and Cr^6+^ quenched the fluorescence completely (Figure 8). Na^+^ and Mg^2+^ had no effect on the fluorescence. These results were similar to what was reported by Paik et al.^4^ and Sun et al.^14^ and increased the possibility that metal ions interact with the lone pairs of oxygen^40^ and thereby disrupt the delocalized electron cloud formed due to the clustering of PEG molecules.

Controlled quenching experiments on 5 nM 3-BHA in water–ethanol (1:1 volume ratio) exhibited loss of fluorescent intensity across all metal ions (Table S1). While the partial quenching occurred at various degrees for Na^+^, Mg^2+^ Co^2+^, and Cr^3+^, a complete quenching was observed for Cr^6+^ and Fe^3+^ for 3-BHA (Table S1). The partial quenching of 3-BHA was significantly more than what was observed for PEG 20k. These results, although not completely confirmatory, indicate that the origin of fluorescence could very well be the 3-BHA fluorophore trapped in the network of PEG clusters. However, the absence of any effect by Na^+^ and Mg^2+^ and a minor impact due to Co^2+^ and Cr^3+^ on the fluorescence intensity in PEG 20k increases the possibility of emission due to aggregation of the PEG matrix, which might involve delocalized electrons interacting “through space” within the PEG 20k aggregates.

### Through-Space Delocalization in PEG 20k

#### 1D-^1^H NMR to Confirm Electron Delocalization

**Figure 9:**
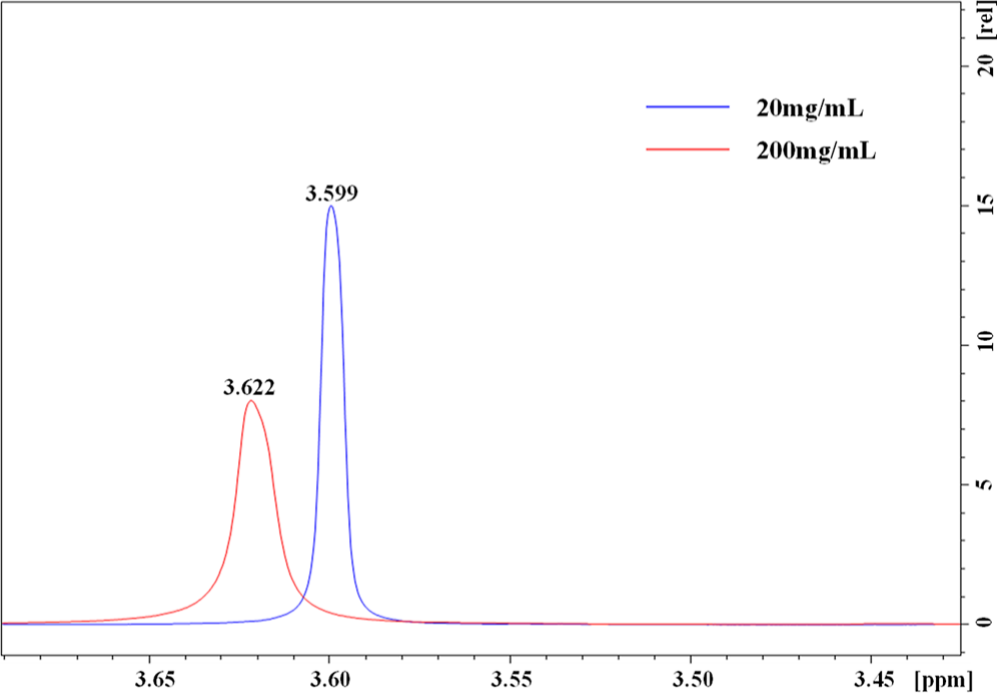
^1^H NMR spectra of PEG 20k in D_2_O at different concentrations.

To further investigate the effect of through-space delocalization, ^1^H NMR of PEG 20k in D_2_O was recorded. ^1^H NMR analysis of PEG 20k in D_2_O at different concentrations revealed a chemical shift at 3.599 ppm for the ethylene (−CH_2_CH_2_−) protons for 20 mg/mL PEG 20k solution. The NMR peak for the methylene protons shifted toward the low field with the increase in the PEG 20k concentration; the peak for methylene protons appeared at 3.622 ppm for PEG 20k at a concentration of 200 mg/mL (Figure 9). This notable shift (∼0.023 ppm) toward the low field supports the fact that the electron density around the −CH_2_– protons was decreasing with increased PEG 20k concentration. The effect of electron-withdrawing groups, i.e., the ether O atoms, on the chemical shift of −CH_2_– protons is due to the cluster formation at higher concentrations. The cluster formation resulted in “through-space” delocalization. A similar observation was reported earlier for PEG 8k.^14^

Although most of the experimental techniques used indicated the presence of PEG 20k clusters in an aqueous solution, which could lead to AIE, none of them confirmed that PEG 20k fluoresces around 300–400 nm. Hence, atomistic simulations and quantum calculations of molecular models were carried out to explore the structure of PEG 20k and examine the possibilities of AIE due to “through-space” delocalization.

### Molecular Models of PEG

#### MD Simulation of PEG 20k Aggregates

**Figure 10:**
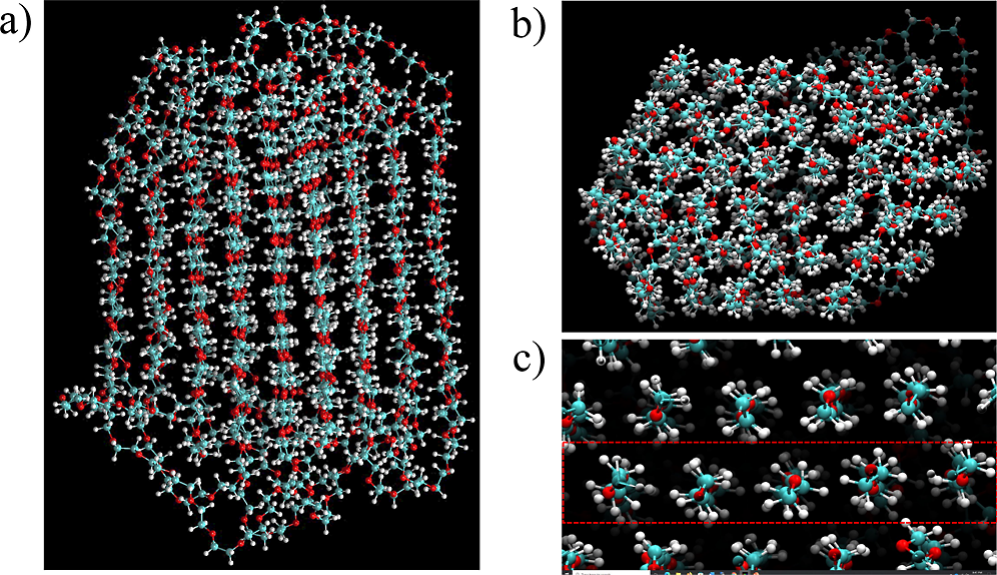
Folded structure of PEG 20k, which was obtained after the gas-phase folded structure was immersed in water and then simulated for 100 ns. The following structures, displayed through VMD, represent planes of the folded PEG 20k—(a) the side view, (b) the top view, and (c) the zoomed-in top view of the planes.

**Figure 11:**
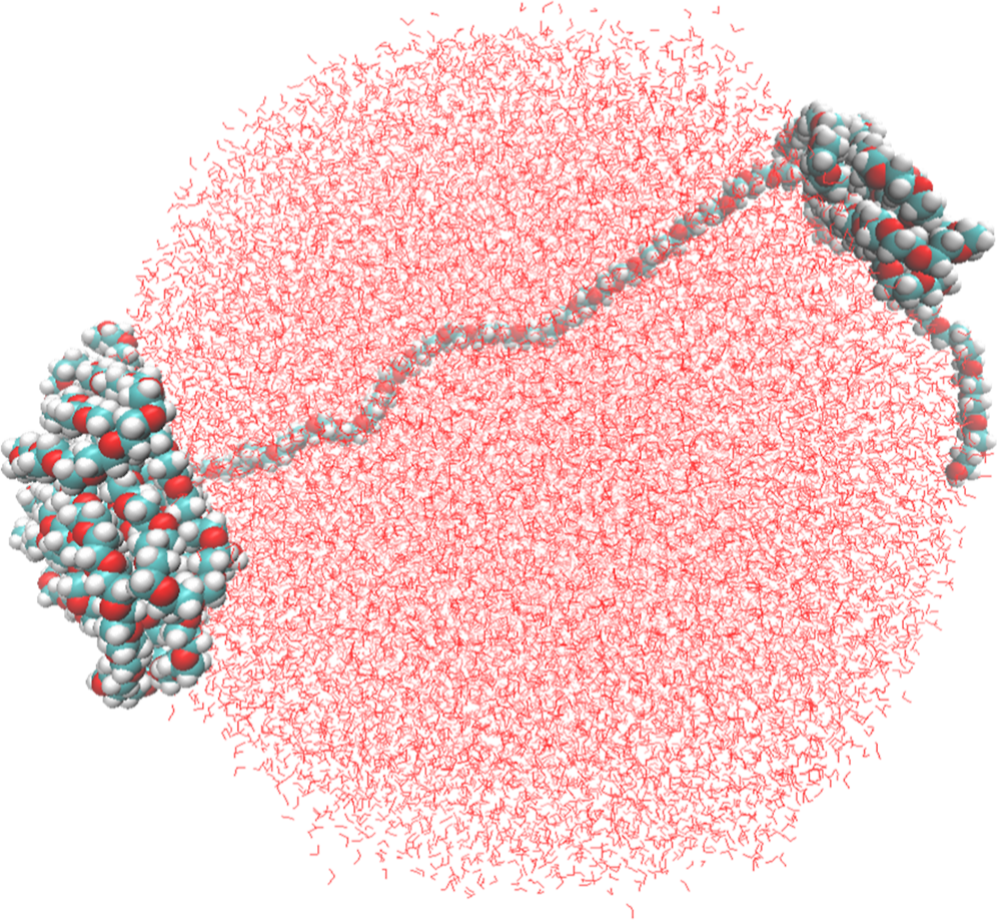
Simulated partially folded PEG 20k molecule in the water sphere.

At first, atomistic MD simulations were performed to investigate the structure of PEG 20k in an aqueous environment. Three types of MD simulations were conducted: a single-folded PEG 20k molecule in the aqueous phase, a partially folded single PEG 20k molecule with water in a spherical boundary, and a heptamer of PEG 20k in the gas phase and aqueous phase. At first, the PEG 20k polymer was built and the structure was minimized, which was then immersed into a water sphere. The initial ball-shaped conformation of the PEG 20k, obtained from the gaseous simulation, began to organize into hexagonal columns when solvated to mimic experimental conditions and simulated for 100 ns (Figure 10a,b). It is also important to note that the interior of the folded structure after 100 ns simulation did not contain any water molecules (Figure 10c). In contrast, when a single PEG 20k chain was allowed to fold in water, it formed clusters at the two ends of the chain, which then floated over the spherical water (Figure 11). This clustering phenomenon suggested that the hydrophobic interactions between the methylene groups of PEG 20k are stronger than the hydrophilic interactions with water molecules. Moreover, hydrophobic clustering also favors the delocalization of the lone pairs of electrons on oxygen atoms.^41^

**Figure 12:**
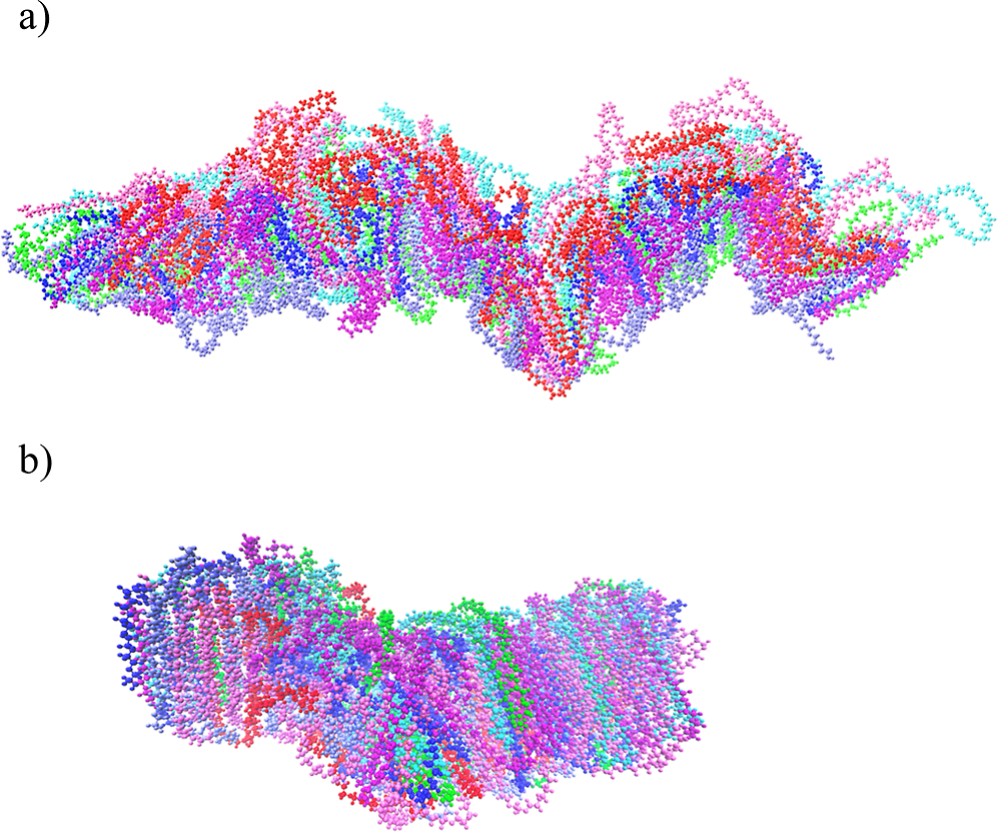
Organized structures of heptameric PEG 20k after 200 ns of gaseous simulation (a) followed by 200 ns of an aqueous simulation (b). These structures are displayed and rendered through VMD. The varying colors represent individual PEG 20k molecules.

The initial gaseous PEG 20k heptamer simulation was used to examine interactions between PEG 20k molecules (intermolecular interactions) and those within a molecule (intramolecular interactions). After 200 ns simulation, each of the seven PEG 20k chains exhibited interactions, not only intramolecular but also between the PEG 20k molecules. Additionally, the structure became much more compact (Figure 12a). To investigate the potential impacts of water on the interactions between PEG 20k molecules, the final structure of the gaseous simulation was solvated at a neutral pH and then simulated for another 200 ns. An extension of the MD simulation time yielded a more compact and organized structure (Figure 12b) than previously seen in the first 200 ns. Similar to a single PEG 20k simulation, the large clusters of the PEG 20k heptamer did not contain any water molecules at the core of the organized cluster, which possessed both inter- and intramolecular interactions.

#### Electronic Structure Calculations of PEG 20k

The quantum chemical nature of the intramolecular interactions within the interior region of the folded PEG 20k was further investigated in small clusters of radii 5, 7.5, and 10 Å, with 70, 223, and 499 number of atoms, respectively (Table 2). In addition, a monomer and a dimer of EG were also used.

**Table 2:** HOMO–LUMO Energy Gap (in kcal/mol) in EG and PEG Clusters of Varied Sizes

molecule	number of atoms	HOMO–LUMO energy gap (kcal/mol)
EG (monomer)	10	230.4
EG (dimer)	19	221.6
5 Å sphere	70	201.8
7.5 Å sphere	223	194.1
10 Å sphere	499	188.2

The M06-2X functional was chosen for all calculations as this functional is parametrized to model the dispersion interactions that arise due to the electron correlational effect.^42^ Furthermore, previous studies demonstrated that the M06-2X functional provides a reliable measure of energetics and geometry with a delocalized electron system.^17,18^

**Figure 13:**
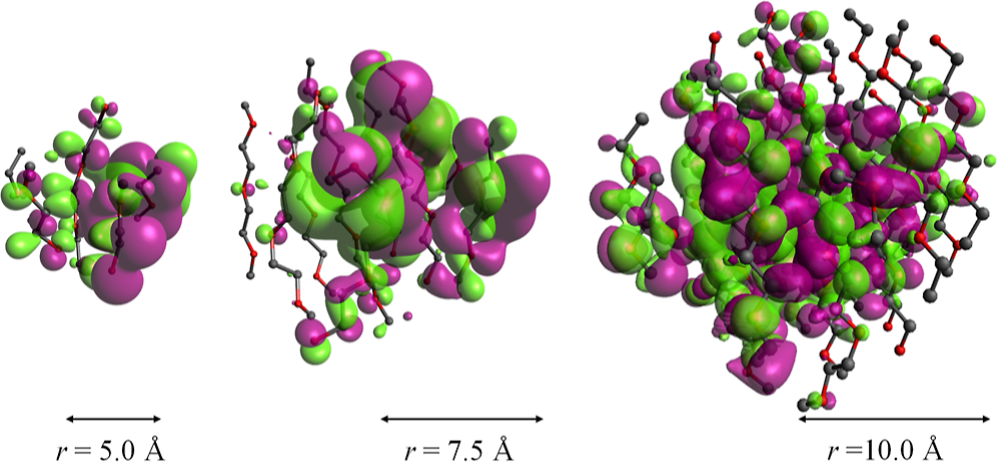
Delocalization of the electron cloud as observed through electronic structure calculation of clusters of variable sizes, extracted from a folded PEG 20k polymer. The HOMO in each cluster is shown; the purple and green colors represent the positive and negative amplitudes, respectively, of the molecular orbital’s wavefunction. Hydrogen atoms are omitted for clarity.

**Figure 14:**
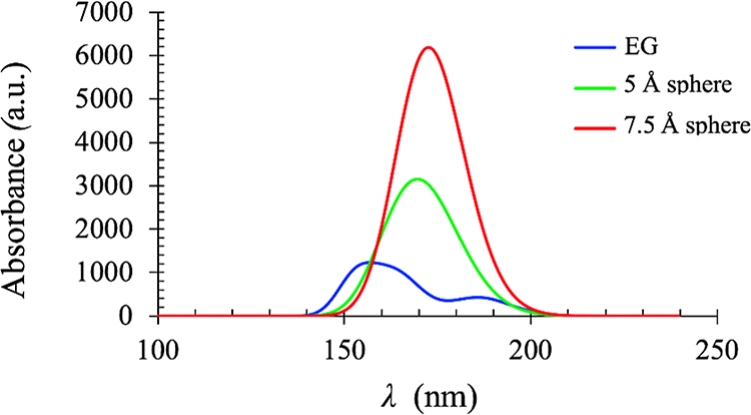
Simulated electronic spectra of EG (monomer) and PEG 20k clusters computed using time-dependent density functional theory.

The electronic structure calculations revealed ordered arrangements of atoms with average interatomic distances of C–C, O–O, and C–O ranging from 3.4 to 4.0 Å between the adjacent columns. As the size of the clusters increased, these interatomic distances remained invariant. However, as the size increased, the highest occupied molecular orbital (HOMO) of these clusters appear to spread over a larger block of atoms, demonstrating increased delocalization (Figure 13). An increase in delocalization with the increase in the cluster size is further established by the decrease in the HOMO–LUMO energy gap (Table 2). A subsequent computation of the electronic transition using TD-DFT confirmed the ground-state calculation results, as described above. The excited-state calculation showed a single peak at 155 nm for the monomeric EG. As discussed in the work of Ueno et al., this peak arises due to the *n*-to-Rydberg orbital transition.^40^ Our computation showed that as the size increased, the peak was red-shifted to 175 nm; this demonstrated more delocalization of the electron density (Figure 14) upon an increase in cluster size. The computed models did not exhibit any absorbance beyond 200 nm, and hence, the study conclusively demonstrated that the core of the PEG 20k aggregate has no absorbance in the 280–300 nm range.

#### Analysis of Partial Charges of Oxygen

The analysis of partial charges of oxygen atoms with the increasing size of PEG 20k clusters portrayed a picture supporting the delocalization. As evident from the data provided in Table 3, there appears to be a discrete change in the partial charges as clusters formed, regardless of their size. The geometry-optimized structures of the EG monomer and its dimer (side-by-side) produced partial charges of ∼−0.8 for oxygen atoms.

**Table 3:** Partial Charge Distribution of Oxygen in EG and PEG Clusters of Varied Sizes^a^

molecule	percentage of atoms carrying an average partial charge of −0.85 or higher	percentage of atoms carrying an average partial charge of −0.65 or higher
EG (monomer/dimer)	100	0
5 Å sphere	44	56
7.5 Å sphere	14	86
10 Å sphere	17	83

a The larger delocalization resulted in the reduced negative charge on oxygen atoms.

In contrast, for all clusters, a maximum partial charge for oxygen atoms of approximately −0.6 occurred (Table 3). As the cluster size increased, the percentage of oxygen atoms carrying diminished negative partial charges increased too, indicating increased delocalization.

## Conclusions

A detailed study, which involved experiments and computations, was conducted to investigate the origin of fluorescence in PEG 20k. Within the range of 280–300 nm excitation wavelength, the emission intensity of PEG 20k is maximum at ∼325 nm for λ_ex_ ∼ 290 nm. Also, PEG 20k emission intensity increased with the increase in concentrations. In the aqueous state, the fluorescence intensity was maximum for PEG 20k compared to other PEG molecules when excited at 295 nm. The lack of fluorescence from lower molecular weight PEG molecules under the present experimental condition, which were earlier reported to exhibit fluorescence properties, prompted us to thoroughly investigate the origin of fluorescence in PEG 20k. We first checked the presence of any fluorescent molecules as impurities in the PEG 20k samples. The GC–MS experiments revealed the presence of 3-BHA in the commercially available PEG 20k. Removal of 3-BHA from PEG 20k using diethyl ether resulted in a significant decrease in the fluorescence intensity compared to the fluorescence intensity of the unpurified PEG 20k. This observation suggested that the emission observed between 300 and 400 nm from the aqueous solution of unpurified PEG 20K is mainly due to 3-BHA. Although the 3-BHA was not completely removed from the PEG 20k sample (there was ∼5% residual 3-BHA present), the fluorescence experiment does not exclude the possibility of PEG 20k behaving as a non-traditional fluorophore.

Various experimental studies suggested that the PEG 20k could form clusters in aqueous solution via intermolecular and intramolecular interactions within and between PEG 20k chains. The formation of clusters, which favors electron delocalization, was observed by AFM imaging and size measurements through DLS. Also, the fluorescent anisotropy measurement revealed the existence of clusters of PEG 20k at higher concentrations at 25 °C; PEG 20k clusters dissociate at a higher temperature (50 °C). This cluster formation could favor the “through-space” delocalization of lone pairs of electrons and could make this non-traditional fluorophore fluoresce via “aggregation-induced emission”. However, the melting temperature experiment and effects of metal ions were inconclusive regarding AIE. Nonetheless, NMR analyses of 20 and 200 mg/mL demonstrated a chemical shift of the ethylene protons (−CH_2_CH_2_−) toward the low field, from 3.599 to 3.622 ppm, suggesting the “through-space” delocalization due to cluster formation.

MD simulations of the PEG 20k monomer and heptamer in the gas phase and the aqueous phase also showed the existence of well-organized hexagonal columns in the folded polymers. These folded polymers were devoid of water molecules at the core, and the folding was favored due to hydrophobic interactions, intra- and intermolecular, between the methylene carbons. The hydrophobic clustering enabled electron delocalization among the organized oxygen atoms with lone pairs and is expected to induce the fluorescent property in PEG 20k. The electronic structure calculations of variable sizes of clusters, extracted from the core of a folded PEG 20k polymer, revealed ordered arrangements of hexagonal columns, where interatomic distances, i.e., C–C, O–O, and C–O, remained independent of cluster size. As the cluster size increased, electron clouds spread over a larger region, and the degree of delocalization increased. The electronic structure calculations provided a clear picture of the lone pairs of electron delocalization and the extended through-space delocalization. The computation of electronic transitions using TD-DFT revealed a single peak due to *n*-to-Rydberg orbital transition, which was red-shifted from 155 to 175 nm with increasing cluster size (Figure 14). However, the UV–Vis spectra of commercially available PEG 20k in water revealed the absorption maxima at ∼220 and ∼290 nm (Figure S4), which matched the reported UV–vis spectra of 3-BHA.^43^ This observation suggested that although PEG 20k forms clusters at higher concentrations, which favors “through-space” delocalization of lone pairs of electrons, there is no evidence that it absorbs in the 280–300 nm range. In contrast, 3-BHA is the main chromophore that absorbs at 290 nm and emits between 300 and 400 nm. Taken together, the observed absence of fluorescent properties of the smaller PEG molecules and the lack of absorption of the quantum chemically simulated PEG 20k core at the excitation wavelength used in earlier studies^4,14^ —despite “through-space” delocalization — strongly suggested that PEG molecules do not fluoresce between 300–400 nm . Furthermore, the presence of the fluorogenic 3-BHA in the commercially available PEG 20k could be deceptive regarding the fluorescence observed for PEG molecules, irrespective of their sizes. Therefore, the reported fluorescent properties^4,14^ of variable-sized PEG molecules should be re-examined.

## Supporting Information Available

Comparative decrease in fluorescence due to metal ions for PEG 20k vs 3-BHA, emission spectra of PEG 20k and PEG of different molecular weights, AFM images, and UV–vis spectrum of PEG 20k (PDF)

## Abbreviations

AFM: atomic force microscopy
AIE: aggregation-induced emission
DLS: dynamic light scattering
EG: ethylene glycol
HOPG: highly oriented pyrolytic graphite
PEG 20k: polyethylene glycol 20,000
MD: molecular dynamics
^1^H NMR: proton nuclear magnetic resonance
