# Supplementary material for: Polyethylene Glycol 20k. Does It Fluoresce?
Source: ACS Omega. 2023 Apr 6;8(15):14208–18. doi: 10.1021/acsomega.3c01124 (PMC10168656; doi:10.1021/acsomega.3c01124)

03/20/2023

Polyethylene Glycol 20k. Does it Fluoresce?

Bethany F. Laatsch[1], Michael Brandt[1], Brianna Finke[1], Carl J. Fossum[1], Miles J. Wackett[1], Harrison R. Lowater[1], Alex Narkiewicz-Jodko[1], Christine N. Le[1], Thao Yang[1], Elizabeth M. Glogowski[2], Scott C. Bailey-Hartsel[1], Sudeep Bhattacharyya[1*], and Sanchita Hati[1*]

[1]Department of Chemistry and Biochemistry, University of Wisconsin-Eau Claire, Wisconsin 54701, U. S. A.

[2]Department of Materials Science and Biomedical Engineering, University of Wisconsin-Eau Claire, Wisconsin, 54701, U. S. A.

[*]To whom correspondence should be addressed: S.B.: phone: 715-836-2278; fax: 715-836-4979; email: bhattas@uwec.edu; S.H.: phone: 715-836-3850; fax: 715-836-4979; email: hatis@uwec.edu

Table S1. Percent decrease of the fluorescent intensity due to metal ions observed in the PEG 20k versus 3-BHA (5 nM). The concentration of all metal ions solution was maintained at 10 mM.

**Metal ions**	**Percentage decrease in PEG 20k**	**Percentage decrease in 3-BHA**
**Na^{+}**	0	93
**Mg^{2+}**	0	94
**Co^{2+}**	0	70
**Cr^{3+}**	7	82
**Fe^{3+}**	100	100
**Cr^{6+}**	100	100

Figure S1. Emission spectra of 100 mg/mL PEG 20k in water.

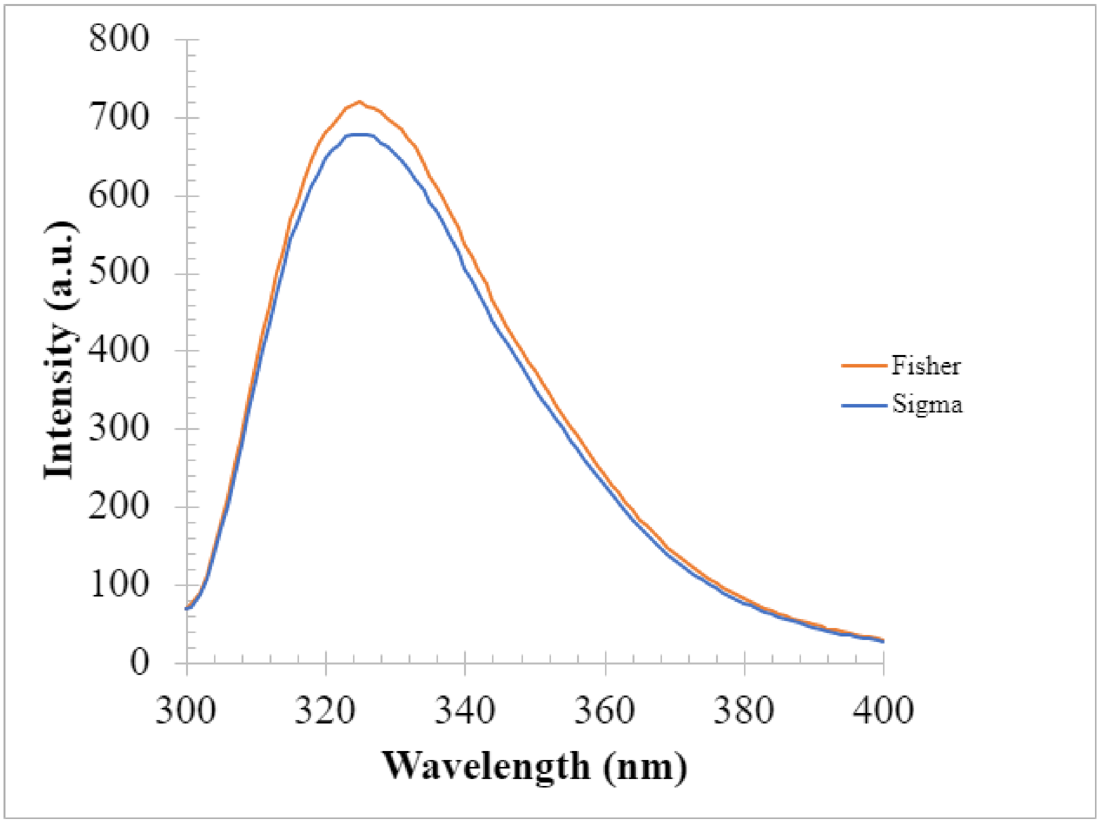

Figure S2. Emission spectra of 100 mg/mL PEG of different molecular weights in water alone over a range of excitation wavelengths, 210-300 nm.

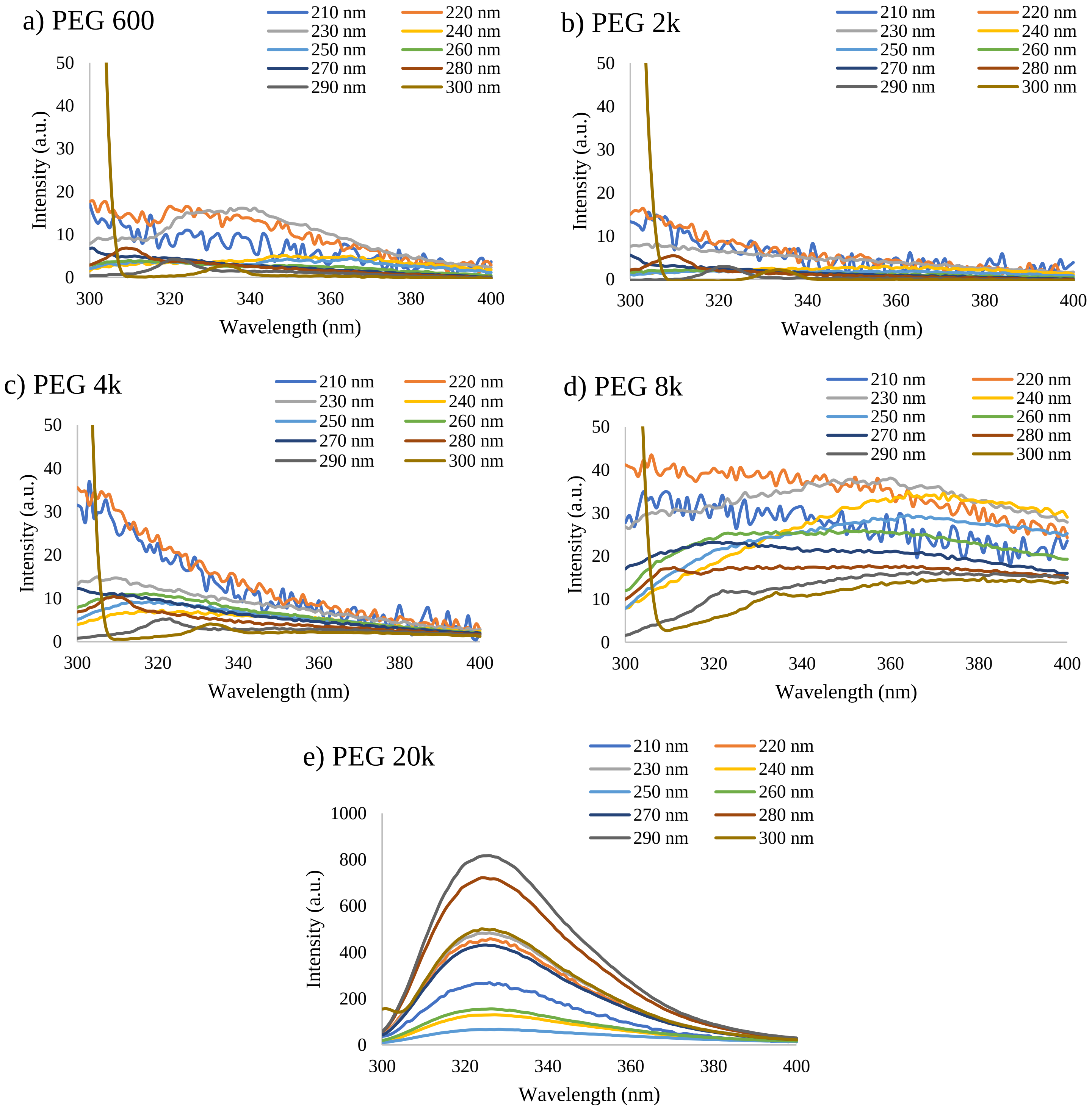

Figure S3. AFM images (height-retraced) of the pure HOPG substrate. a) AFM image size is 5 μm x 5 μm; the key indicates a length of 1 μm. b) AFM image size is 0.45 μm x 0.45 μm; the key indicates a length of 450 nm.

a) HOPG Substrate - 5 micron

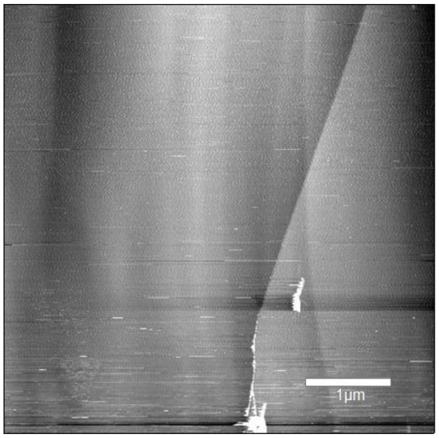

b) HOPG Substrate - Zoomed-in on image (a), 0.45 micron

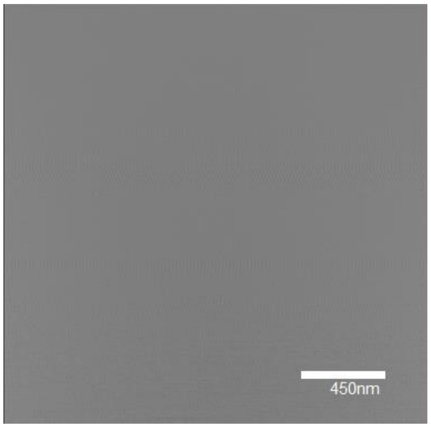

Figure S4. UV-Vis spectra of 30 mg/mL and 300 mg/mL of PEG 20k in water.

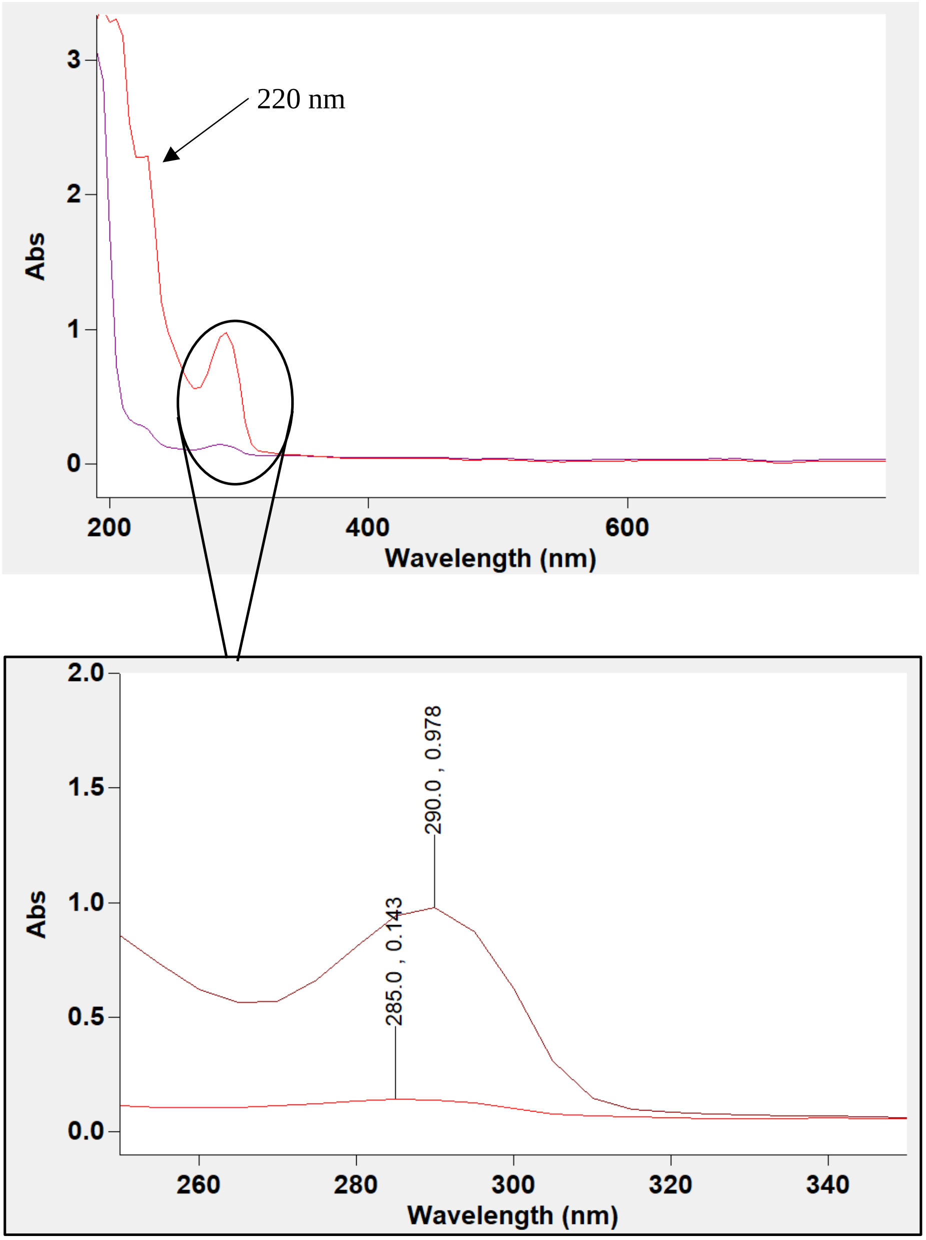

Supplement: Supplementary file 1 — ao3c01124_si_001.pdf [file ao3c01124_si_001.pdf]
